# The Impact of Aerobic Exercise on Fronto-Parietal Network Connectivity and Its Relation to Mobility: An Exploratory Analysis of a 6-Month Randomized Controlled Trial

**DOI:** 10.3389/fnhum.2017.00344

**Published:** 2017-06-30

**Authors:** Chun L. Hsu, John R. Best, Shirley Wang, Michelle W. Voss, Robin G. Y. Hsiung, Michelle Munkacsy, Winnie Cheung, Todd C. Handy, Teresa Liu-Ambrose

**Affiliations:** ^1^Aging, Mobility, and Cognitive Neuroscience Lab, University of British Columbia, VancouverBC, Canada; ^2^Department of Physical Therapy, University of British Columbia, VancouverBC, Canada; ^3^Djavad Mowafaghian Center for Brain Health, University of British Columbia, VancouverBC, Canada; ^4^Center for Hip Health and Mobility, VancouverBC, Canada; ^5^Health, Brain, and Cognition Lab, University of Iowa, Iowa CityIA, United States; ^6^Department of Psychology, University of Iowa, Iowa CityIA, United States; ^7^Department of Medicine, University of British Columbia, VancouverBC, Canada; ^8^Department of Psychology, University of British Columbia, VancouverBC, Canada

**Keywords:** aging, impaired mobility, vascular cognitive impairment, fronto-parietal network, functional connectivity, fMRI

## Abstract

Impaired mobility is a major concern for older adults and has significant consequences. While the widely accepted belief is that improved physical function underlies the effectiveness of targeted exercise training in improving mobility and reducing falls, recent evidence suggests cognitive and neural benefits gained through exercise may also play an important role in promoting mobility. However, the underlying neural mechanisms of this relationship are currently unclear. Thus, we hypothesize that 6 months of progressive aerobic exercise training would alter frontoparietal network (FPN) connectivity during a motor task among older adults with mild subcortical ischemic vascular cognitive impairment (SIVCI)—and exercise-induced changes in FPN connectivity would correlate with changes in mobility. We focused on the FPN as it is involved in top-down attentional control as well as motor planning and motor execution. Participants were randomized either to usual-care (CON), which included monthly educational materials about VCI and healthy diet; or thrice-weekly aerobic training (AT), which was walking outdoors with progressive intensity. Functional magnetic resonance imaging was acquired at baseline and trial completion, where the participants were instructed to perform bilateral finger tapping task. At trial completion, compared with AT, CON showed significantly increased FPN connectivity strength during right finger tapping (*p* < 0.05). Across the participants, reduced FPN connectivity was associated with greater cardiovascular capacity (*p* = 0.05). In the AT group, reduced FPN connectivity was significantly associated with improved mobility performance, as measured by the Timed-Up-and-Go test (*r* = 0.67, *p* = 0.02). These results suggest progressive AT may improve mobility in older adults with SIVCI via maintaining intra-network connectivity of the FPN.

## Introduction

Impaired mobility is a major concern for older adults and is associated with increased risk for disability, institutionalization, and death (Rosano et al., 2008). The prevalence of impaired mobility is 14% at age 75 years and involves half of the population over 84 years (Odenheimer et al., 1994). Falls are a significant consequence of impaired mobility.

Current evidence supports the recommendation of targeted exercise training to improve mobility, prevent major mobility disability, and reduce the risk of future falls (Campbell et al., 1999; Pahor et al., 2014). The widely accepted view is that improved physical function, such as improved balance and increased muscle strength, primarily underlies the effectiveness of the exercise in improving mobility and reducing falls (Layne et al., 2017). However, in a meta-analysis of four randomized trials of exercise, falls were significantly reduced by 35% while postural sway significantly improved by only 9% and there was no significant improvement in knee extension strength (Robertson et al., 2002). Moreover, in a proof-of-concept randomized controlled trial, we demonstrated that a home-based exercise signficantly reduced falls by 47% in older adults – in the absence of significant improvement in physical function (i.e., balance and muscle strength) (Liu-Ambrose et al., 2008). Rather, significant improvement in executive functions were observed in the exercise group as compared with the usual care (i.e., control) group.

These data suggest that exercise may reduce falls in older adults via several mechanisms, not just via improved physical function.

We previously proposed that cognitive and neural plasticity may be an important, yet under-appreciated mechanism by which exercise promotes mobility and reduce falls (Liu-Ambrose et al., 2013). This hypothesis stems from the growing evidence that suggest: (1) cognitive impairment and impaired mobility are associated (Atkinson et al., 2007; Buracchio et al., 2010; Montero-Odasso et al., 2012); (2) reduced executive function, is associated with impaired mobility and increased falls risk (Delbaere et al., 2012; Hsu et al., 2012); (3) aberrant neural network functional connectivity is associated with impaired mobility (Hsu et al., 2014); and (4) targeted exercise training, particulary aerobic-based, promotes cognitive and cortical plasticity, including executive function and its neural correlates, in older adults (Erickson and Kramer, 2009; Voss et al., 2010b).

Despite the growing recognition that targeted exercise training may promote mobility outcomes in older adults via central mechanisms (Liu-Ambrose et al., 2013), few intervention studies of exercise to date have provided direct evidence for this theory (Bolandzadeh et al., 2015). A better understanding of the neural mechanisms underlying exercise-induced improvements in mobility may facilitate the development and refinement of preventative/intervention strategies, as well as identify the populations for whom these effects apply.

Older adults with subcortical ischemic vascular cognitive impairment (SIVCI), the most common form of vascular cognitive impairment (VCI) (Bowler, 2005), are at particular risk for both impaired mobility and dementia secondary to underlying white matter lesions (WMLs) or lacunar infarcts (Roman et al., 2002; Vermeer et al., 2007). VCI is the second most common cause of dementia after Alzheimer’s disease (AD) (Desmond et al., 1999; Erkinjuntti et al., 1999; Pantoni et al., 1999; Rockwood et al., 2000). The clinical consequences of covert ischemic strokes are substantial (Roman et al., 2002; Vermeer et al., 2007). These WMLs and lacunar infarcts typically manifest in brain regions such as caudate, pallidum, thalamus, frontal and prefrontal white-matter (Roman et al., 2002). As a result, they may disrupt the integrity of functional neural networks and negatively impact cognitive function, particulary executive functions, and mobility (Kuo and Lipsitz, 2004; O’Brien, 2006).

Among the relevant neural networks, most notably, is the frontoparietal network (FPN). The FPN is involved in top-down attentional control and allocation of available neural resources that contribute to executive processes, such as response anticipation and conflict processing (Fogassi and Luppino, 2005; Seeley et al., 2007; Sridharan et al., 2008), as well as motor planning and motor execution (Wise et al., 1997; Wu et al., 2009; Wymbs et al., 2012). Of particular relevance, previous studies have shown that key regions within the FPN were actively recruited during actual as well as imagined completion of the walking while talking (WWT) test (Holtzer et al., 2011; Blumen et al., 2014). Specifically, neural activity within these FPN regions were positively associated with both task difficulty and cognitive performance of the WWT test (Holtzer et al., 2011; Blumen et al., 2014). Although previous studies have linked aspects of mobility to FPN connectivity, its potential role understanding exercise-induced effects on mobility is unkown.

Thus, we propose FPN connectivity as one of the neural mechanisms by which exercise promotes mobility in older adults with mild SIVCI. Using functional magnetic resonance imaging (fMRI) data from a 6-month single-blind randomized controlled trial (clinicaltrials.gov Identifier: NCT01027858), we conducted a planned secondary analysis to assess the impact of moderate-intensity aerobic exercise training on functional connectivity of FPN among older adults with mild SIVCI. We hypothesized that aerobic exercise-induced increases in FPN connectivity would correlate with improved mobility. The primary results from the parent study have been published (Liu-Ambrose et al., 2016), which provided preliminary evidence that 6 months of thrice-weekly progressive aerobic training (AT) promotes cognitive performance in community-dwelling adults with mild SIVCI, relative to usual care plus education.

## Materials and Methods

### Study Design

This is a secondary analysis of neuroimaging data acquired from a 6-month proof-of-concept RCT (NCT01027858) of aerobic exercise in older adults with mild SIVCI (Liu-Ambrose et al., 2010, 2016). Trained study assessors were blinded to group allocation of participants. Functional MRI (fMRI) data were acquired at baseline prior to randomization and at trial completion (i.e., 6 months) in a subset of eligible participants.

### Participants

As the current study was a secondary analysis, we sought to recruit as many eligible and consenting individuals from the parent study as possible. To briefly describe the recruitment process, we recruited from the University of British Columbia Hospital Clinic for Alzheimer’s Disease and Related Disorders, the Vancouver General Hospital Stroke Prevention Clinic, and specialized geriatric clinics in Metro Vancouver, BC. Recruitment occurred between December 2009 and April 2014 with randomization occurring on an ongoing basis. Study participants were clinically diagnosed with mild SIVCI as determined by the presence of cognitive syndrome and small vessel ischaemic disease (Erkinjuntti et al., 2000). Small vessel ischemic disease was defined as evidence of relevant cerebrovascular disease by brain computed tomography or MRI defined as the presence of both: (1) Periventricular and deep WMLs; (2) Absence of cortical and/or cortico-subcortical non-lacunar territorial infarcts and watershed infarcts, hemorrhages indicating large vessel disease, signs of normal pressure hydrocephalus, or other specific causes of WMLs (i.e., multiple sclerosis, leukodystrophies, sarcoidosis, brain irradiation). In addition to the neuroimaging evidence, the presence or a history of neurological signs such as Babinski sign, sensory deficit, gait disorder, or extrapyramidal signs consistent with sub-cortical brain lesion(s) was required and confirmed by study physicians (G-YRH and PL). Cognitive syndrome was defined as a baseline Montreal Cognitive Assessment (MoCA) score less than 26/30. However, participants were free of frank dementia (i.e., clinically diagnosis of dementia) as determined by a Mini-Mental State Examination (MMSE) score ≥ 20 and the absence of diagnosed dementia. Progressive cognitive decline was confirmed through medical records or caregiver/family member interviews.

**FIGURE 1:**
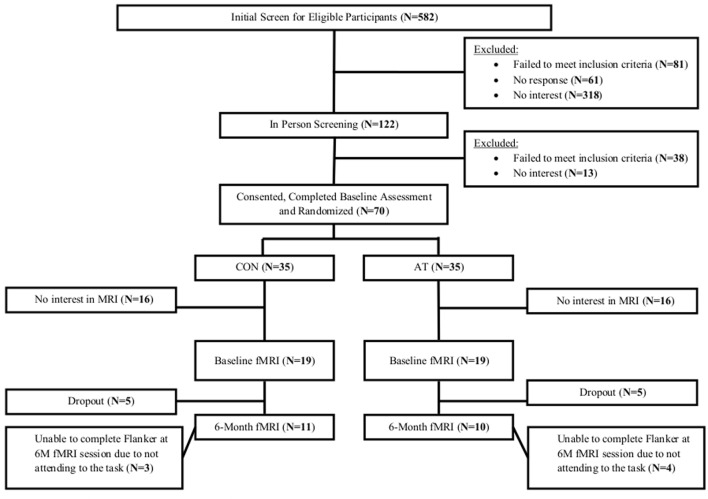
Overview of the flow of study participants through the 6-month study.

The Consolidated Standards of Reporting Trial flowchart shows the number and distribution of participants included in this secondary analysis (**Figure 1**). Of the 38 participants (54% of parent sample) that completed baseline MRI scanning, 7 (18% of the sample) dropped out from the study and 10 (26% of the sample) failed to correctly perform the motor finger tapping task (e.g., finger tapped during resting blocks). Consequently, 21 participants who completed MRI at baseline and trial completion were included in this secondary analysis (30% of parent sample). Ethical approval was provided by the University of British Columbia’s Clinical Research Ethics Board (H07-01160). All participants provided written informed consent.

### Randomization

The randomization sequence was generated using the web application www.randomization.com with a ratio of 1:1 to AT or usual care (CON). A research team member not involved with the study held this sequence at a remote location. After the completion of consent and baseline testing, the research coordinator contacted the team member holding the list to determine the next allocation.

### Aerobic Training and Compliance

For the AT group, AT consisted of supervised thrice-weekly 60-min classes of walking for the 6-month intervention period. All AT group classes were led by instructors certified to instruct seniors and were delivered in a group setting. Each 60-min class included a 10-min warm-up, 40-min of walking, and a 10-min cool down. Both the warm-up and cool-down included passive and active stretches, as well as range of motion exercise.

Walking occurred outdoors and followed predetermined routes around local areas. The intensity of the AT program was monitored and progressed using three approaches: (1) heart rate monitoring with an initial intensity of 40% of age specific target heart rate (i.e., heart rate reserve; HRR). HRR was calculated by subtracting resting heart rate from maximum heart rate [using the formula: 206.9 – 0.67 × Age (Gellish et al., 2007)] and recalculation each month. Participants progressed over the first 12 weeks to the range of 60–70% of HRR, after which this was sustained for the remainder of the intervention period; (2) subjective monitoring using the Borg’s Rating of Perceived Exertion (RPE) (Borg, 1982) with a target RPE of 14 to 15; and (3) the “talk” test (Persinger et al., 2004), starting at a walking pace allowing comfortable conversation and progressing to a walking pace where conversation was difficult. Individual training logs (i.e., target heart rate, heart rate achieved, and rate of perceived exertion) were maintained throughout the intervention period.

The AT group was also given a pedometer to serve as both an incentive and monitoring tool. Participants recorded the number of steps each day taken outside the AT classes on standard logs provided by the research team.

### Usual Care

Participants in the CON group received usual care, in which they were provided with monthly educational materials about VCI and healthy diet. However, no specific information regarding physical activity was provided. In addition, research staff phoned the CON participants on a monthly basis to maintain contact and to acquire research data.

### Adverse Effects

All participants were instructed to report any adverse effects due to the AT exercises to our research coordinator, such as falls or musculoskeletal pain persisting longer than 48 h. Participants were also questioned about the presence of any adverse effects, such as musculoskeletal pain or discomfort, at each exercise session. All instructors also monitored participants for symptoms of angina and shortness of breath during the exercise classes. External experts from our safety monitoring committee reviewed all adverse events reported on a monthly basis.

### Descriptive Variables

At baseline, participants underwent a clinical assessment with study physicians (GYRH and PL) to confirm current health status and study eligibility. Age in years and education level were assessed by self-report. Standing height was measured as stretch stature to the 0.1 cm per standard protocol. Weight was measured twice to the 0.1 kg on a calibrated digital scale. Waist-to-hip ratio was determined by measuring the widest part of the hip circumference and the waist just above the navel in centimeters. The Functional Comorbidity Index (Groll et al., 2005) assessed the number of comorbid conditions related to physical functioning.

Global cognition was assessed using the MMSE (Cockrell and Folstein, 1988) and the MoCA. The MMSE and MoCA are 30-point tests that encompass several cognitive domains. The MoCA has been found to have good internal consistency and test–retest reliability and was able to correctly identify 90% of a large sample of individuals with mild cognitive impairment from two different clinics with a cut-off scores of ≤26/30 (Nasreddine et al., 2005).

### Functional MRI Acquisition

All MRI was conducted at the University of British Columbia (UBC) MRI Research Center located at the UBC Hospital on a 3.0 Tesla Intera Achieva MRI Scanner (Philips Medical Systems, Markham, ON, Canada) using an 8-channel SENSE neurovascular coil. The fMRI consisted of two successive runs with 165 dynamic images of 36 slices (3 mm thick) with the following parameters: repetition time (TR) of 2000 ms, echo time (TE) of 30 ms, flip angle (FA) of 90 degrees, field of view (FoV) of 240 mm, acquisition matrix 80 × 80, voxel size of 3 mm × 3 mm × 3 mm. High resolution anatomical MRI T1 images were acquired using the following parameters: 170 slices (1 mm thick), TR of 7.7 ms, TE of 3.6 ms, FA of 8 degrees, FoV of 256 mm, acquisition matrix of 256 × 200.

During each scanning session, the study participants were asked to perform a finger tapping motor task that had been previously administered and described (Hsu et al., 2014). Briefly, the task consisted of three conditions: right finger tapping, rest, and left finger tapping. The specific instructions given required the participants to finger tap in a particular sequence regardless of condition: start with the index finger and progress toward the little (pinky) finger continuously until a different condition is presented. For the rest condition, participants were asked to rest with their eyes open. The exact order of motor task blocks was not disclosed to the participants and was counter balanced over two runs as follow:

Run A: Rest, Left Tap, Rest, Right Tap, Rest, Right Tap, Rest, Left Tap, Rest
Run B: Rest, Right Tap, Rest, Left Tap, Rest, Left Tap, Rest, Right Tap, Rest

Total WML volume (in mm^3^) at baseline was quantified with structural MRI data acquired on the same MRI scanner (3T Achieva, Philips Medical Systems, Markham, ON, Canada) at the UBC MRI Research Centre. A T2-weighted scan and a proton-density-weighted (PD-weighted) scan were acquired for each subject. For the T2-weighted images, the repetition time (TR) was 5,431 ms and the echo time (TE) was 90 ms, and for the PD-weighted images, the TR was 2,000 ms, and the TE was 8 ms. T2- and PD-weighted scans had dimensions of 256 × 256 × 60 voxels and a voxel size of 0.937 mm × 0.937 mm × 3.000 mm. Briefly, WMLs were identified and digitally marked (i.e., placing seed points) by a radiologist on T2 and PD weighted images. Marked WMLs were automatically segmented by a customized Parzen windows classifier that estimated the intensity distribution of the lesions – which also included heuristics that optimized the accuracy of the estimated distributions (Parzen, 1962; McAusland et al., 2010; Bolandzadeh et al., 2015). WML segmentation was reviewed by a trained technician to ensure accuracy.

### Mobility, Cardiovascular Capacity, and Physical Activity

Mobility was assessed with the Timed-Up-and-Go test (TUG) and the Short Physical Performance Battery (SPPB). The TUG required participants to rise from a standard chair, walk a distance of three meters, turn, walk back to the chair and sit down (Shumway-Cook et al., 2000). We recorded the time (s) to complete the TUG, based on the average of two separate trials.

For the SPPB, participants were assessed on performances of standing balance, walking, and sit-to-stand. Each component is rated out of four points, for a maximum of 12 points; a score < 9/12 predicts subsequent disability (Guralnik et al., 1995).

Participant’s cardiovascular capacity was assessed using the 6-Minute Walk Test (Enright, 2003). The total distance walked (meters) within the span of 6 min was recorded.

Monthly total physical activity level was determined by the Physical Activities Scale for the Elderly (PASE) self-report questionnaire (Washburn et al., 1999).

### Data Analysis

#### Functional MRI Preprocessing

Image preprocessing was carried out using tools from FSL (FMRIB’s Software Library) [78], MATLAB (Matrix Laboratory), and toolboxes from SPM (Statistical Parametric Mapping). Excess unwanted structures (i.e., bones, skull, etc.) in high resolution T1 images were removed via Brain Extraction Tool (BET); rigid body motion correction was completed using MCFLIRT (absolute and relative mean displacement were subsequently extracted and included in the statistical analysis as covariates); spatial smoothing was carried out using Gaussian kernel of Full-Width-Half-Maximum (FWHM) 6.0 mm; temporal filtering was applied with high pass frequency cut-off of 120 s. In addition, a low pass temporal filtering was also included to ensure the fMRI signal fluctuated between 0.008 < f < 0.080 Hz, the ideal bandwidth to examine functional connectivity. Furthermore, the application of a low pass filter eliminated high frequency signals that could be confounds. Participants’ low-resolution functional data were registered to personal high resolution T1 anatomical images, which were subsequently registered to standardized 152 T1 Montreal Neurological Institute (MNI) space.

Noise generated from both physiological and non-physiological sources were removed through regression of the cerebral-spinal fluid (CSF) signal, white matter signal, and global brain signal. Global signal regression had been reported as both valid and useful step in functional connectivity analyses (Fox et al., 2009) that may improve specificity (Murphy and Fox, 2016).

#### Functional Connectivity Analysis

Previous studies guided our choice of seeds in the whole brain analysis of the FPN (Voss et al., 2010b). The FPN included the inferior parietal sulcus (IPS), ventral visual cortex (VV), supramarginal gyrus (SMG), superior lateral occipital cortex (SLOC), frontal eye field (FEF), as well as overlapping areas in the temporal-parietal junction. The respective MNI space coordinates for each region of interest (ROI) are provided in **Table 1**.

**Table 1:** Frontoparietal network regions of interest and relative MNI coordinates.

FPN	ROI	*X*	*Y*	*Z*
	RIPS	25	-62	53
	RVV	36	-62	0
	LVV	-44	-60	-6
	RSMG	32	-38	38
	RSLOC	26	-64	54
	LSLOC	-26	-60	52
	RFEF	28	-4	58
	LFEF	-26	-8	54


*RIPS, right inferior parietal sulcus; RVV, right ventral visual; LVV, left ventral visual; RSMG, right supramarginal gyrus; RSLOC, right lateral occipital cortex; LSLOC, left lateral occipital cortex; RFEF, right frontal eye field; LFEF, left frontal eye field.*

From each ROI, preprocessed time-series data were extracted with 14 mm spherical regions of interest drawn around their respective MNI coordinates in standard space. The different conditions (i.e., left, right, and rest) within each block of the motor task were extracted and compiled together. To concatenate the time-series data, the stimulus onset time for each task condition was acquired from the task program. Each volume of the data was then sorted according to their respective condition. Once the data were properly categorized, the task-specific volumes (e.g., all the “left” volumes) were merged using a script provided in the FSL program. The first three volumes of any condition were discarded to account for delay of the hemodynamic response. Evidence in the literature has demonstrated that functional connectivity derived from temporally spliced/merged resting-state data from blocked fMRI design is not significantly different from connectivity derived from continuous data (Fair et al., 2007). Recent study using motor task fMRI also showed that quantifying functional connectivity via similar seed-based approach using concatenated data is comparable to results from continuous data (Zhu et al., 2017).

Region of interest time-series data were subsequently cross-correlated with every voxel within the brain to establish functional connectivity maps of their associated neural networks, in which pairwise correlation between time-series extracted from ROI listed above was calculated. Individual-level within-subject results were generated via ordinary least-squares (OLS) regression using FSL’s flameo (Beckmann et al., 2003) in FSL by congregating the voxel-wise functional connectivity maps from each condition. Similarly, for group results, a mixed-level OLS analysis was conducted. The statistical map thresholding was set at *Z* = 2.33, with cluster correction of *p* < 0.05.

#### Statistical Analyses

Statistical analysis was conducted using the IBM SPSS Statistic 23 for Windows (SPSS Inc., Chicago, IL, United States). Statistical significance was set at *p* ≤ 0.05 for all analyses. Change in network connectivity strength was computed in SPSS as 6-month FPN connectivity minus baseline FPN connectivity. Linear mixed models with random intercepts and time-varying outcome measures were constructed to statistically test for significant between-group differences in change in network connectivity while adjusting for baseline total WML and age. A group by time interaction indicated group differences in changes in FPN connectivity from baseline to post-intervention. Similar analyses were conducted to determine whether there were group differences in changes in TUG, SPPB, 6MWT, and PASE scores. The primary analyses included the 21 participants with valid baseline and post-intervention fMRI data. Secondary analyses followed the intention-to-treat principle by including nine additional individuals with valid baseline fMRI but were lost to follow-up; maximum likelihood estimation allowed for these individuals to inform the treatment effects, despite having missing follow-up data and to determine whether loss to follow-up might bias the treatment effects estimated with only treatment completers.

Bivariate correlation analyses were performed to determine whether any significant changes in intra-network FPN connectivity (during rest, left tap, and right tap) in the AT group (*n* = 12) correlated with change in mobility, as measured by TUG and SPPB, or change in 6MWT across the 6-month study duration.

## Results

### Participants and Treatment Fidelity

Among the 70 randomized individuals in the parent study, we observed a significant effect of AT on 6-min walk performance, a well-established tool that accurately evaluates cardiovascular fitness (Cataneo et al., 2010) (*B* = 30.34, *p* = 0.02), indicating that AT had a positive effect on cardiovascular capacity (Liu-Ambrose et al., 2016). Twenty-one participants who completed fMRI scans at both baseline and 6 months were included in the primary analysis (**Figure 1**). Study demographics are reported in **Table 2**, pedometer information over the intervention period is reported in **Table 3**, mobility and cardiovascular capacity measures are reported in **Table 4**; these measures do not differ between groups at baseline nor differ significantly from the 70 eligible participants enrolled in the parent study (Liu-Ambrose et al., 2016). The mean age of all participants included in this secondary analysis was 71.1 years (*SD* = 8.7 years), which is not significantly different from the mean age of the parent cohort at 74.3 years (*SD* = 8.3 years). Compared to the nine individuals with valid baseline data only, the study sample did not differ on baseline FPN connectivity (all *p* > 0.19) but did have higher average baseline MoCA scores (23.2 versus 20.4; *p* = 0.02). Neither of the mobility measures nor self-reported physical activity differed significantly between groups across the 6 months. However, we observed a trend-level group difference in the change in 6-Minute Walk Test performance (*p* = 0.08; **Table 4**), in which the AT group showed greater improvement (48.6 m) compared with the CON group (-0.3 m).

**Table 2:** Participant characteristics at baseline (*N* = 21).

	CON group, *n* = 9	AT Group, *n* = 12
Variables	Mean (*SD*) or *n*	Mean (*SD*) or *n*
Age (year)	69.9 (9.2)	72.0 (8.6)
Height (cm)	165.4 (11.4)	169.0 (15.9)
Weight (kg)	73.6 (13.1)	73.6 (14.8)
Sex (M/F)	5/4	8/4
MMSE (30 points max)	27.2 (1.9)	26.4 (2.8)
MOCA (30 points max)	24.2 (2.3)	22.5 (2.0)
FCI	3.0 (1.9)	3.1 (1.7)
White matter lesion (mm^3^)	1389.5 (2023.4)	3492.8 (3882.1)
Relative head motion (mm)	0.17 (0.09)	0.14 (0.07)


*MMSE, Mini-Mental Status Examination; MoCA, Montreal Cognitive Assessment; FCI, Functional Comorbidity Index. All measures were not statistically significant at *p* < 0.05.*

**Table 3:** AT group pedometer information over 6-month intervention period.

	Average pedometer count
	Mean (*SD*)
Baseline	7002.1 (5496.4)
Month 1	9009.2 (6337.9)
Month 2	9786.3 (6944.2)
Month 3	10713.8 (7209.0)
Month 4	10922.1 (7645.7)
Month 5	11484.8 (8074.6)
Month 6	11552.7 (7411.9)


**Table 4:** Mobility and cardiovascular capacity measures (*N* = 21).

	Baseline		Change from baseline to month 6^∗^ (end of intervention)		
		
	Mean (SD)		Adjusted within-group change (SE)		
			
Variables	CON	AT	CON	AT	Between-group *p*-value
Timed Up and Go (s)	8.2 (1.3)	7.6 (1.8)	0.16 (0.46)	-0.06 (0.41)	0.73
Short Physical Performance Battery (max 12 points)	10.4 (0.9)	11.2 (1.3)	0.48 (0.34)	-0.23 (0.31)	0.15
Six-Minute Walk Test (meters)	531.2 (56.9)	555.6 (104.3)	-0.30 (19.29)	48.64 (16.57)	0.08
PASE Score	132.2 (61.3)	134.6 (94.0)	-6.05 (39.62)	-4.99 (34.05)	0.99


*^∗^Adjusted for baseline WML and age; PASE, Physical Activities Scale for the Elderly.*

### AT Compliance and Adverse Effects

The average compliance observed in the AT group was 76% for the walking classes and 65% for the nutrition education classes; whereas the average compliance observed in the CON group was 74%. Two study-related adverse events were reported in the AT group and one in the CON group. All three were non-syncopal falls. One of the falls in the AT group resulted in a broken tooth and required assessment in the Emergency Department; the remaining two did not result in injury.

### fMRI Results

**FIGURE 2:**
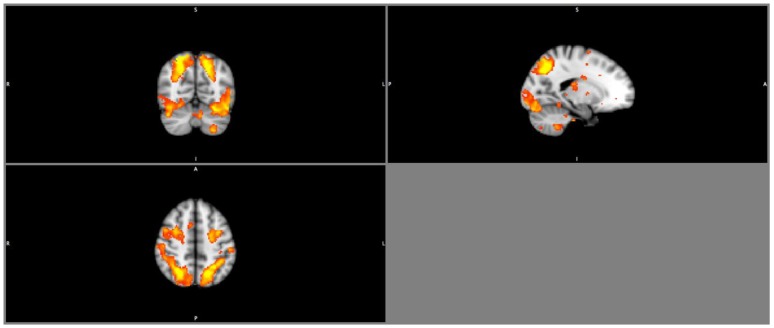
Image of the fronto-parietal network.

Results from the seed-based functional connectivity analysis on the FPN (**Figure 2**) showed there were no significant between group differences in the mean network connectivity strength at baseline, regardless of task conditions (**Table 5**). At trial completion, compared with AT, CON exhibited significantly greater intra-network coupling of the FPN during right finger tapping (*p* < 0.02) after adjusting for baseline WML and age. No AT effects were observed for FPN connectivity during left finger tapping (*p* = 0.26) or during rest (*p* = 0.50). We conducted a secondary, intention-to-treat analysis using all 30 participants with usable baseline data, regardless of loss to follow-up, and observed similar, though weaker, between-group differences in FPN coupling during right finger tapping (*p* = 0.08). As with the primary analyses, CON showed an increase in intra-network coupling of the FPN (mean = 0.18, *SE* = 0.09), whereas AT showed no significant change over time (mean = -0.04, *SE* = 0.08).

**Table 5:** Frontoparietal network connectivity during task (*N* = 21).

	Baseline		Change from baseline to month 6^∗^ (end of intervention)		
		
	Mean (SD)		Adjusted within-group change (SE)		
			
Task condition	CON	AT	CON	AT	Between-group *p*-value
Right Tapping	0.25 (0.28)	0.34 (0.22)	0.25 (0.09)	-0.06 (0.07)	0.02
Rest	0.29 (0.24)	0.26 (0.24)	0.06 (0.09)	-0.02 (0.07)	0.50
Left Tapping	0.35 (0.24)	0.27 (0.20)	-0.05 (0.08)	0.08 (0.07)	0.26


*^∗^Adjusted for baseline WML and age.*

### Correlation Results

**FIGURE 3:**
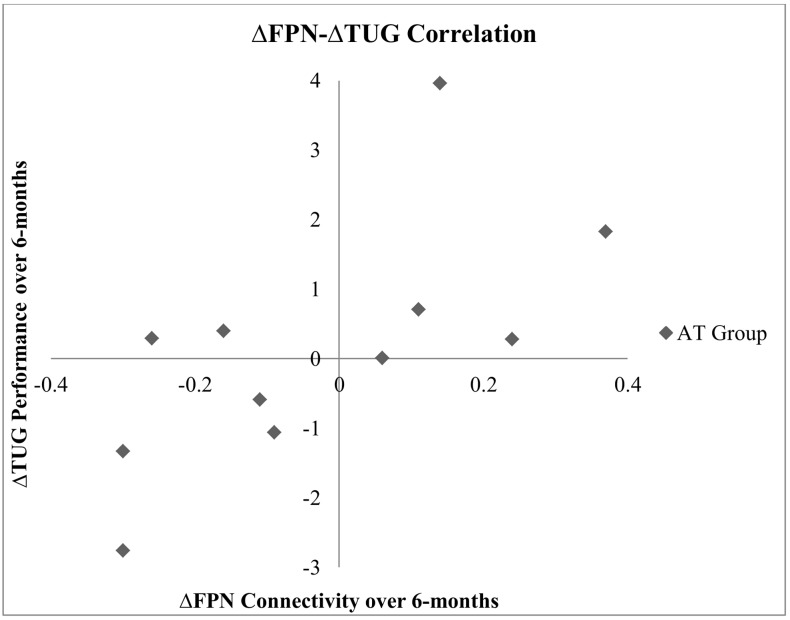
Correlation between change in TUG performance and change in FPN connectivity during right finger tapping within the AT group.

Bivariate correlation across the study sample showed that the change in FPN connectivity during right tapping was significantly associated with change in 6-Minute Walk Test performance (*r* = -0.43, *p* = 0.05; **Table 6**). Within the AT group (*N* = 12), the change in FPN connectivity during right tapping was significantly associated with change in TUG performance (*r* = 0.67, *p* = 0.02; **Table 6**). Specifically, reduced FPN connectivity from baseline to post-intervention correlated with improved TUG performance over the same period of time (**Figure 3**).

**Table 6:** Changes in mobility and changes in FPN connectivity correlation.

	ΔTimed	ΔShort physical	ΔSix-minute
	up and go	performance battery	walk test
Group (*N* = 21)			
ΔFPN Connectivity during right tapping	0.14	0.34	-0.43^∗^
AT group (*n* = 12)			
ΔFPN Connectivity during right tapping	0.67^∗^	0.14	-0.37


*Δ = 6-Months – Baseline. ^∗^*p* < 0.05.*

## Discussion

Contrary to our initial hypothesis, we found that a 6-month AT intervention significantly alters FPN connectivity during right finger tapping among older adults with mild SIVCI. The observed effect of aerobic exercise on the FPN during right tapping was significantly associated with improved mobility and cardiovascular capacity. While these results are preliminary, our data suggest aerobic exercise may promote mobility among older adults with mild SIVCI via altering FPN connectivity.

Our findings are in contrast to previous findings that show altered FPN connectivity is associated with aging (Andrews-Hanna et al., 2007) and with cognitive deficits (He et al., 2007; Geerligs et al., 2012; Poppe et al., 2015). Specifically, Poppe et al. (2015) demonstrated that compared with healthy controls, patients with schizophrenia had significantly less connectivity in the FPN during goal-oriented task performance. Compared with controls, task performance was also significantly worse among patients. Similarly, He et al. (2007) found that compared with age-matched healthy controls, individuals who suffered an acute stroke showed significantly less left–right posterior intraparietal sulcus connectivity while performing a spatial-orientation task. Lower connectivity between the left and right posterior intraparietal sulcus were significantly associated with poorer task accuracy and slower task reaction time.

Moreover, current evidence suggests that SIVCI is generally associated with less functional connectivity of neural networks. For example, among those with SIVCI, Yi et al. (2012) found lower functional connectivity in the medial prefrontal cortex and the middle temporal gyrus. Yu et al. (2015) demonstrated that compared with healthy controls, individuals with SIVCI had less network efficiency in the fronto-temporal and parietal regions. Nonetheless, aberrant functional connectivity has been repeatedly observed among those with SIVCI (Yi et al., 2012; Ding et al., 2015; Yu et al., 2015; Zhou et al., 2016), and the pattern (i.e., increased or decreased connectively) is not consistent across studies. For instance, Ding et al. (2015) found increased functional connectivity in the left middle temporal lobe, right inferior temporal lobe, and left superior frontal gyrus among patients with SIVCI as compared with healthy controls.

However, our results do concur with and extend emerging evidence that show less functional connectivity of large-scale networks may be advantageous (Chuang et al., 2014), especially within the context of mobility. In one cross-sectional study, Rosenberg-Katz et al. (2015) demonstrated that compared with healthy older adults and individuals with Parkinson’s disease who were non-fallers, those with Parkinson’s disease who were fallers showed significantly greater connectivity between the posterior parietal lobule and the inferior parietal lobule. This data suggest increased connectivity between parietal regions may be associated with more severe motor impairments and more generally, heightened neural activity (e.g., activation or connectivity) may reflect the inability of networks to actively suppress irrelevant neural events, causing regions to compete unnecessarily for available neural resources. In contrast, diminished connectivity may represent greater efficiency as the networks can effectively allocate resources to areas of immediate importance. Certainly, emerging evidence suggests that lifestyle interventions can improve neural efficiency (Smith et al., 2013; Nishiguchi et al., 2015).

Our observation that AT impacted FPN connectivity only during right hand finger tapping concurs with the literature that suggests the FPN connectivity is lateralized (Smith et al., 2009; Pool et al., 2014; Gao et al., 2015). Specifically, using independent component analysis, Smith et al. (2009) revealed that among the neural networks identified, only the FPN exhibit distinct left-right lateralized components. Also, Jancke et al. (2000) found contra-lateralized FPN activation during right index finger tapping task (without visual cue), including the left dorsal lateral premotor cortex and the left inferior parietal lobule. Moreover, Yuan et al. (2015) recently demonstrated that gait velocity among cognitively normal older adults was significantly associated with connectivity of the left-FPN. Therefore, given that our study participants were all right-hand dominant, our results are supported by the literature. We also extend the current state of knowledge by using data generated from a randomized controlled trial to demonstrate the potential impact of aerobic exercise on FPN connectivity during right hand finger tapping and the significant association between FPN connectivity and mobility and cardiovascular capacity. An alternative interpretation of these results may be that aerobic exercise may help maintain mobility and cardiovascular capacity among older adults with SIVCI via reducing cognitive load (i.e., less FPN connectivity) required to perform less attention-demanding motor task (i.e., dominant hand finger tapping). Our own previous work supports this latter concept (Hsu et al., 2017). Critically, in this separate sub-analysis of the same 6-month RCT, we found that after AT, older adults confirmed with SIVCI performed significantly better at the Eriksen flanker task compared with the no-exercise controls. The observed improvement in task performance was associated with overall reduction in activation in the lateral occipital cortex and superior temporal gyrus.

It should be noted that we are aware of only one study in the relevant field in the literature that investigated the association between functional connectivity and cardiorespiratory fitness among older adults (Voss et al., 2010a). While our findings deviate from evidence presented, in which the authors reported greater connectivity is associated with higher fitness among healthy older adults (Voss et al., 2010a), several distinctions from the current study should be considered. Specifically, the differences were: (1) the fMRI task (visual vs. motor); (2) the network examined (default mode network vs. FPN); and (3) study participant (healthy older adults vs. older adults with SIVCI). The combination of these variations could have resulted in disparities in the reported findings.

A few limitations should be taken into consideration. First, our study participants are likely healthier and to have superior physical functioning than average older adults with mild SIVCI. This potential sample bias is somewhat unavoidable given the requirement that participants be able to engage in progressive AT safely. However, it also limits the generalizability of our findings to the population of older adults with mild SIVCI as a whole. Secondly, due to the small sample size of the current study, the current dataset may not possess enough power to detect small differences between the two groups. Provided that the study population is generally frail and older, the occurrence of drop-out from potentially strenuous fMRI session is to be expected. Future studies designed with larger sample sizes are necessary to validate the notion of functional network efficiency/inefficiency by providing sufficient power despite the expectation of drop-out. Thirdly, it is possible that subsets of pairwise connectivity between ROI within the FPN may have driven the effects we observed; however, this was not further investigated due to potential issue with type II error with the current sample size. Moreover, there is much controversy in regards to global signal regression and potential observation of artificial anti-correlations. This may be particularly influential when examining functional connectivity between networks deemed anti-correlated in nature (e.g., default mode network and FPN). In assessing within-network connectivity, it may be that the effects of induced anti-correlation are less significant. However, as stated by Murphy and Fox (Murphy and Fox, 2016) ‘there is not a single “right” way to process resting state data that reveals the “true” nature of the brain.’ They also summarized the several advantages of global signal regression including removal of motion, cardiac and respiratory signals. In addition, despite evidence supporting its use (Fair et al., 2007; Zhu et al., 2017), we recognize temporally splicing and concatenating data is not recommended and can potentially lead to increase in signal noise. Nevertheless, studies demonstrated that connectivity derived from concatenation does not differ significantly from those acquired from continuous data (Fair et al., 2007; Zhu et al., 2017). In addition, our data is limited by the fact that only the connectivity during right hand tapping was statistically significant while left hand was not. Differences in social interactions experienced by the experimental groups may present addition confounding factors to our data. Specifically, active attention provided by trainers within the AT group may potentially influence our findings. Lastly, the relationship between connectivity and SIVCI status is equivocal with much of the evidence generated from cross-sectional studies. Thus, the inclusion of fMRI data from a healthy-aged matched cohort might have facilitated interpretation of our results. Nevertheless, we highlight the key strengths of our currents study design – a randomized controlled trial – which are: (1) provides evidence of causation; and (2) increased internal validity. Thus, our study provides preliminary evidence to suggest that aerobic exercise may impact functional connectivity in older adults with SIVCI, and this is associated with the maintenance of mobility.

## Conclusion

Our results demonstrate that neural network functional connectivity may contribute to the effects of aerobic exercise on mobility among older adults with SIVCI. We observed that 6 months of AT maintains motor task-based connectivity within the FPN of older adults with SIVCI, and the degree of decoupling within this region correlates with improvements in mobility. As such, our current findings support emerging results from others that altered functional connectivity within certain neural networks might represent a beneficial change in older adults with mild SIVCI, especially vis-à-vis their mobility. More broadly, these results bring further support to the burgeoning notion that functional neural changes contribute to exercised-induced improvements to mobility among older adults. As extension of these findings, future studies should explore potential interaction between mobility and cognitive outcomes among this population.

## Data Access and Responsibility

TL-A had full access to all the data in the study and takes responsibility for the integrity of the data and the accuracy of the data analysis.

## Author Contributions

TL-A and RH were involved in the study concept, design, acquisition of data, preparation and critical review of the manuscript. CH, MM, WC, and TL-A were involved in data collection. CH, TL-A, and JB were involved in writing of the manuscript. CH, TL-A, and JB were involved in statistical analyses. CH, TL-A, and JB were involved in interpretation of data. CH and SW were involved in fMRI data analyses. MV and TH were involved in critical review of the manuscript. All authors had full access to all of the data (including statistical reports and tables) in the study and can take responsibility for the integrity of the data and the accuracy of the data analysis.

## Conflict of Interest Statement

The authors declare that the research was conducted in the absence of any commercial or financial relationships that could be construed as a potential conflict of interest. The reviewers KM, GA and handling Editor declared their shared affiliation, and the handling Editor states that the process nevertheless met the standards of a fair and objective review.
