# Corrigendum: The Impact of Aerobic Exercise on Fronto-Parietal Network Connectivity and Its Relation to Mobility: An Exploratory Analysis of a 6-Month Randomized Controlled Trial

**DOI:** 10.3389/fnhum.2017.00449

**Published:** 2017-09-05

**Authors:** Chun L. Hsu, John R. Best, Shirley Wang, Michelle W. Voss, Robin G. Y. Hsiung, Michelle Munkacsy, Winnie Cheung, Todd C. Handy, Teresa Liu-Ambrose

**Affiliations:** ^1^Aging, Mobility, and Cognitive Neuroscience Lab, University of British Columbia Vancouver, BC, Canada; ^2^Department of Physical Therapy, University of British Columbia Vancouver, BC, Canada; ^3^Djavad Mowafaghian Center for Brain Health, University of British Columbia Vancouver, BC, Canada; ^4^Center for Hip Health and Mobility Vancouver, BC, Canada; ^5^Health, Brain and Cognition Lab, University of Iowa Iowa City, IA, United States; ^6^Department of Psychology, University of Iowa Iowa City, IA, United States; ^7^Department of Medicine, University of British Columbia Vancouver, BC, Canada; ^8^Department of Psychology, University of British Columbia Vancouver, BC, Canada

**Keywords:** aging, impaired mobility, vascular cognitive impairment, fronto-parietal network, functional connectivity, fMRI

In the original article, we used inconsistent wording that, while not incorrect, may cause confusion for readers. In the discussion section and the conclusion, we indicated that we found aerobic training may alter functional network connectivity. For greater clarity, we should have stated that aerobic training maintains network connectivity.

The following corrections, in italics, have been made to Discussion, Paragraph 1.

Contrary to our initial hypothesis, we found that a 6-month AT intervention *did not significantly increase, but rather maintained* FPN connectivity during right finger tapping among older adults with mild SIVCI. The observed effect of aerobic exercise on the FPN during right tapping was significantly associated with improved mobility and cardiovascular capacity. While these results are preliminary, our data suggest aerobic exercise may promote mobility among older adults with mild SIVCI by *maintaining the integrity* of FPN connectivity.

Also, a correction has been made to Conclusion, Paragraph 1.

Our results demonstrate that neural network functional connectivity may contribute to the effects of aerobic exercise on mobility among older adults with SIVCI. We observed that 6 months of AT maintained motor task-based connectivity within the FPN of older adults with SIVCI, and the degree of decoupling within this region correlated with improvements in mobility. As such, our current findings support emerging results from others that *lower* functional connectivity within certain neural networks might represent a beneficial change in older adults with mild SIVCI, especially vis-à-vis their mobility. More broadly, these results bring further support to the burgeoning notion that functional neural changes contribute to exercised-induced improvements to mobility among older adults. As an extension of these findings, future studies should explore potential interactions between mobility and cognitive outcomes among this population.

The authors apologize for this issue and state that this does not change the scientific conclusions of the article in any way.

## Conflict of interest statement

The authors declare that the research was conducted in the absence of any commercial or financial relationships that could be construed as a potential conflict of interest.

